# Supplementary material for: TP53 p.R337H Germline Variant among Women at Risk of Hereditary Breast Cancer in a Public Health System of Midwest Brazil
Source: Genes (Basel). 2024 Jul 16;15(7):928. doi: 10.3390/genes15070928 (PMC11276326; doi:10.3390/genes15070928)
Supplement: Supplementary file 1 [file genes-15-00928-s001.zip › genes-2978195-supplementary.pdf]

genes

MDPI

Supplementary Materials

Table S1: NCCN version 1.2020

Criteria
Age at diagnosis of breast cancer < 45 years
Breast cancer between 46-50 years and an additional breast cancer
Breast cancer between 46-50 years and a close relative with breast cancer at any age on the same side
Triple-negative breast cancer at age , 60 years
One ore more first-, second-, or third-degree relative with breast cancer at age < 50 years on the same side of the family
Breast cancer any age and Family history of ovarian cancer
Breast cancer any age and Family history of male breast cancer
Breast cancer any age and Family history of pancreatic cancer
Breast cancer any age and 2 ore more additional diagnoses of breast cancer in close relatives
Ashkenazi-Jewish ancestry
Personal history of ovarian cancer
Personal history of pancreatic cancer

Table S2: Modified Chompret criteria for TP53 screening

Criteria
**Category A**
The proband developed an LFS core tumor (breast cancer, osteosarcoma, adrenocortical cancer, or brain tumor) < 46 years old and:
-At least one first- or second-degree relative had a history of an LFS core tumor < 56 years old
-If the proband has breast cancer, close relatives with breast cancer should be excluded
The proband has multiple cancers (excluding bilateral breast cancers), two of which are LFS core tumors that first developed < 46 years old
**Category B**
Patients with adrenocortical cancer, choroid plexus cancer, anaplastic rhabdomyosarcoma, Breast cancer patients aged ≤ 31 years, osteosarcoma, childhood hypodiploid acute lymphoblastic leukemia or Sonic Hedgehog Medulloblastoma
Family history is not applicable

Table S3: List of pathogenic variants detected through the NGS panel (among 80 of the 180 patients enrolled)

Gene	Variant	Variant ID (Clinvar)	Number of patients
BRCA1	c.441+2T>A	55196	1
BRCA1	c.5062_5064delGTT (p.Val1688del)	55368	1
BRCA1	c.5074+2T>C	37631	1
BRCA1	c.5266dup (p.Gln1756fs)	17677	1
BRCA2	c.1813dup (p.Ile605fs)	37762	1
BRCA2	c.2808_2811del (p.Ala938Profs)	9322	1
BRCA2	c.3848dup (p.Ser1284fs)	37860	1
BRCA2	c.4005dup (p.Phe1336fs)	51581	1
BRCA2	c.4935del (p.Glu1646fs)	51745	1
BRCA2	c.6405_6409delCTTAA (p.Asn2135Lysfs)	38043	1
BRCA2	c.771_775del (p.Asn257fs)	9326	1
BRCA2	c.9097dup(p.Thr3033fs)	38208	1
TP53	**c.1010G>A (p.Arg337His)**	**12379**	**1**
TP53	c.438G>A (p.Trp146Ter)	428890	1
RET	c.1998G>C (p.Lys666Asn)	230926	1
NTLH1	c.244C>T (p.Gln82Ter)	192319	1
NTLH1	Unspecified	-	1
